# 705 Reduction in Central Line Associated Blood Stream Infection Rate with a Central Line Change-Over Protocol

**DOI:** 10.1093/jbcr/irac012.259

**Published:** 2022-03-23

**Authors:** Jamie L Hollowell, Felicia Williams, Daniel Blandon-Hendrix, Booker King, Rabia Nizamani, Lori Chrisco, Patricia Gebe, Emily Hoke

**Affiliations:** UNC Jaycee Burn Center, Hurdle Mills, North Carolina; UNC Jaycee Burn Center, Chapel Hill, North Carolina; UNC Jaycee Burn Center, Chapel Hill, North Carolina; UNC Jaycee Burn Center, Chapel Hill, North Carolina; UNC Jaycee Burn Center, Chapel Hill, North Carolina; North Carolina Jaycee Burn Center, Chapel Hill, North Carolina; UNC Jaycee Burn Center, Chapel Hill, North Carolina; UNC Jaycee Burn Center, Chapel Hill, North Carolina

## Abstract

**Introduction:**

Burn Intensive Care Units (BICU)s traditionally have changed central lines over a wire (COWs) as a method to reduce infection. To date, there is not a standard timeline for this process and every center has their own timeline for this process. We aimed to create a standardized process for this practice to then have a baseline for future study.

**Methods:**

The Change-Over-Wire (COW) process was evaluated with the BICU Infection Control Nurse and Performance Improvement Manager. A protocol was developed that includes step-by-step detail that was modified until it was deemed practical and acceptable to all involved parties as the best practice for maintaining the most sterility as possible. The protocol was provided to the Central Line team, the ICU Advisory Board, and the UNC CLABSI committee for review

**Results:**

While the hospital as a whole does not standardly endorse routine COWs, the protocol was reviewed and accepted as best practice by both the ICU Advisory Board and the UNC CLABSI committee. Additionally, the BICU had an 80% reduction in CLABSI’s from FY 2017 to 2020 with implementation of this protocol (p value = 0091).

**Conclusions:**

Having a standardized method for COWs both allows for best practice and a starting point for study. Endorsement by both hospital committees allows this practice to be the published standard by which future studies can be measured.

